# Differential predictability of cognitive profiles from brain structure in older males and females

**DOI:** 10.1007/s11357-023-00934-y

**Published:** 2023-09-21

**Authors:** Christiane Jockwitz, Camilla Krämer, Paulo Dellani, Svenja Caspers

**Affiliations:** 1 Institute of Neuroscience and Medicine (INM-1), Research Centre Jülich, Jülich, Germany; 2 Institute for Anatomy I, Medical Faculty & University Hospital Düsseldorf, Heinrich Heine University Düsseldorf, Düsseldorf, Germany

**Keywords:** Aging, Cognitive profiles, Sex differences, Machine learning

## Abstract

Structural brain imaging parameters may successfully predict cognitive performance in neurodegenerative diseases but mostly fail to predict cognitive abilities in healthy older adults. One important aspect contributing to this might be sex differences. Behaviorally, older males and females have been found to differ in terms of cognitive profiles, which cannot be captured by examining them as one homogenous group. In the current study, we examined whether the prediction of cognitive performance from brain structure, i.e. region-wise grey matter volume (GMV), would benefit from the investigation of sex-specific cognitive profiles in a large sample of older adults (1000BRAINS; N = 634; age range 55–85 years). Prediction performance was assessed using a machine learning (ML) approach. Targets represented a) a whole-sample cognitive component solution extracted from males and females, and b) sex-specific cognitive components. Results revealed a generally low predictability of cognitive profiles from region-wise GMV. In males, low predictability was observed across both, the whole sample as well as sex-specific cognitive components. In females, however, predictability differences across sex-specific cognitive components were observed, i.e. visual working memory (WM) and executive functions showed higher predictability than fluency and verbal WM. Hence, results accentuated that addressing sex-specific cognitive profiles allowed a more fine-grained investigation of predictability differences, which may not be observable in the prediction of the whole-sample solution. The current findings not only emphasize the need to further investigate the predictive power of each cognitive component, but they also emphasize the importance of sex-specific analyses in older adults.

**Supplementary Information:**

The online version contains supplementary material available at 10.1007/s11357-023-00934-y.

## Introduction

Structural brain imaging parameters have been related to cognitive decline during the aging process [1]. For example, larger grey matter volume (GMV) of the prefrontal cortex has been associated with better executive functions, while a preserved memory performance was reported to be associated with larger volumes of the medial temporal lobe ([2, 3]; for reviews on brain structure – cognitive performance relation, see [4] and [5]).

Recently, this relationship has become a promising target for the early detection of cognitive deficits in old age. In light of the growing aging population, research has turned to gain a greater understanding of the nature of cognitive decline and impairment during older ages and with it to pave the way for the future amelioration of neurodegenerative diseases. So far, machine learning (ML) approaches have been proven to successfully predict cognitive performance from brain structural imaging data in pathological conditions [6]. For example, Hojjati, Babajani-Feremi [7] have shown that performance on two clinical screening tests, i.e. Clinical Dementia Rating Scale – Sum of boxes and Alzheimer’s Disease Assessment Scale – 13 item version, could be successfully predicted from structural brain features, particularly the volume of the entorhinal cortex and hippocampus, in a large sample of healthy controls (HCs) as well as patients with mild cognitive impairment (MCI) or Alzheimer’s disease (AD) (R^2^ range: 0.54–0.67). Further, it has been demonstrated that structural brain features, e.g. grey matter density, alone or in conjunction with other variables reliably predict memory performance in two large cohorts (i.e. ADNI and DELCOLDE) of HCs, MCI, and AD patients [8, 9]. However, focusing on samples of HC, either across the lifespan or during later decades of life, ML approaches mostly failed to reliably predict cognitive abilities with high accuracies [10, 11].

In search of confounding factors that may conceal brain-phenotype relationships, sex differences in cognitive profiles might be of special interest. Previous studies have not only shown that sex differences in cognitive performance persist until late adulthood but also reported sex imbalances in the prevalence of neurodegenerative diseases, which are accompanied by different cognitive impairments [12–14]. As such, males are more likely to suffer from MCI and Parkinson’s disease, while females are more often affected by Alzheimer’s disease [14, 15]. In a previous study [16], we additionally showed that males and females not only differ in specific cognitive tasks but more generally show specific cognitive profiles across cognitive domains (i.e. attention, executive functions, memory, and language functions). Thereby, cognitive profiles were derived from 16 different cognitive tests as part of an extensive neuropsychological battery in the 1000BRAINS study [17] using an exploratory principal component analysis (ePCA). Results highlighted that cognitive profiles could not be captured well by examining them as one homogenous group. In this context, males displayed a superordinate cognitive system, i.e. an attentional-executive-fluency-memory component, suggesting a stronger interplay of different cognitive functions. Females, on the other hand, exhibited cognitive profiles that consisted of more subsystems as compared to males, with each system including different cognitive functions (i.e. [1] visual (working) memory/ [2] fluency/ [3] executive functions/ [4] verbal (working) memory). Although these functions share covariances, they represent distinct cognitive systems or modules [16]. Thus, it appears that males use a rather integrative cognitive processing system, while cognitive processing in females tends to be more segregated into subsystems.

This observation might be particularly interesting for the prediction of cognitive profiles in older adults. It might not only explain so far only partially successful prediction of cognitive functions in mixed samples of healthy older males and females but also studies addressing sex differences in the predictability of cognitive abilities from imaging data using sex-independent cognitive targets (e.g. theory-driven cognitive components based on mixed results from males and females) [18, 19]. Therefore, the current study examined whether the predictability of cognitive performance from brain structure differs between general cognitive profiles derived from the whole sample and sex-specific cognitive profiles.

## Methods

### Subjects

Subjects included in the current study were drawn from 1000BRAINS [17], a population-based epidemiological cohort study, recruited from the Heinz-Nixdorf Recall study that has been conducted in the Ruhr area in Germany [20]. Since the current study builds on our previous study [16] assessing cognitive profiles in older males and females, we used this sample as starting point of subject selection: 676 subjects between 55 and 87 years matched for age and education (338 males with a mean age of 66.9 years ± 6.7 and a mean ISCED score of 6.3 ± 1.74 and 338 females with a mean age of 66 years ± 6.5 and a mean ISCED score of 6.1 ± 1.86); for exclusion criteria of this study, see [16]). From this sample, 23 males and 19 females had to be excluded due to missing imaging data or methodological problems within the imaging processing pipeline. This resulted in 315 males with a mean age of 67.0 years ± 6.6 and a mean ISCED score of 6.3 ± 1.74 and 319 females with a mean age of 66.1 years ± 6.5 and a mean ISCED score of 6.1 ± 1.86. To verify the matching for age and education, we applied independent samples t-tests, which revealed no significant age and education differences between the two sexes (age: *t*(623) = 1.732, p = 0.084; education: *t*(623) = 1.454, p = 0.146). All participants gave written informed consent before participating in 1000BRAINS. All experiments were performed in accordance with relevant guidelines and regulations. The study protocol was approved by the local Ethics Committee of the University of Essen.

### Sex-specific cognitive profiles 

All subjects underwent intensive neuropsychological testing during their participation in 1000BRAINS including 16 different cognitive functions: selective attention, processing speed, problem solving, concept shifting, susceptibility to interference, figural fluency, phonematic and semantic verbal fluency, vocabulary, verbal episodic memory, figural memory, visual-, visual-spatial- and verbal short-term/working memory. For an overview of cognitive tasks and variables used with mean values as well as sex differences and intercorrelations in test scores, see [16].

**Fig. 1:**
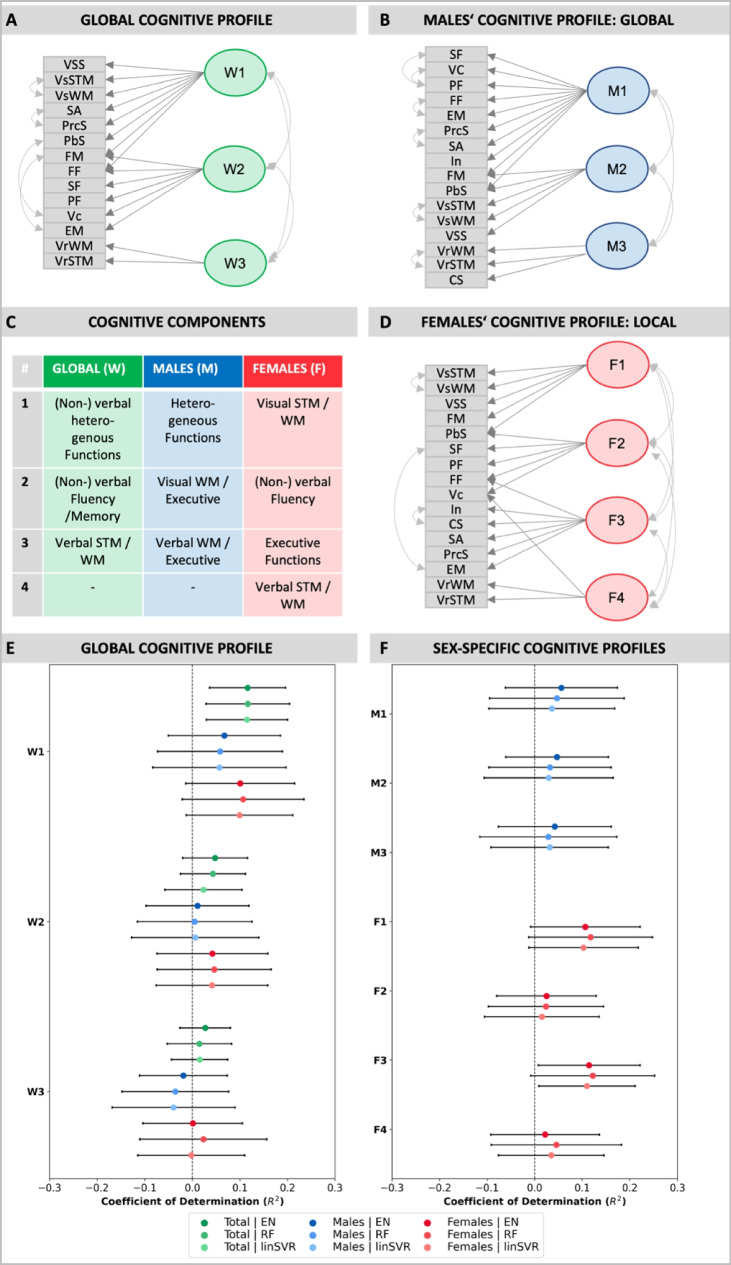
Prediction performance of whole-sample and sex-specific cognitive profiles from region-wise GMV. **A**-**D** Whole sample and sex-specific cognitive component solutions and their loadings are shown. **E**–**F** ML estimations are displayed for the global cognitive profile (left) in the whole sample, in females and males as well as for sex-specific profiles (right) in males and females across different algorithms, i.e. Elastic Net (EN) regression, Random Forest (RF) regression and Linear Support Vector Regression (linSVR). The coefficient of determination (R^2^) is displayed with error bars representing the standard deviation (SD). The grey shaded areas indicate R^2^ > 0.1 suggesting satisfactory prediction performance. Results from no-deconf. condition shown

Based on these cognitive abilities, [16] assessed the research question of whether males and females would show different cognitive profiles, i.e. different compositions of cognitive components. To investigate this, we extracted cognitive components for both, the whole group (n = 676) including males and females, as well as for males (n = 338) and females (n = 338) separately. To do so, we first used an exploratory principal component analysis (ePCA) with Varimax rotation (implemented in the “psych” package, RStudio) to reduce the data into cognitive components. Afterward, a confirmatory factor analysis (CFA, implemented in the “lavaan" package, RStudio) with a maximum likelihood estimator with robust standard errors and a Satorra-Bentler scaled test statistic was applied to validate the different component solutions (for a detailed description of this two-step procedure, see [16], for the final component solutions in the whole group, males and females separately, see Fig. 1A-D). Finally, we extracted regression coefficients for each cognitive component (implemented in the “lavaan” package, RStudio).

### Structural brain imaging

For brain structural analyses, a 3D high-resolution T1-weighted magnetization prepared rapid acquisition gradient-echo (MPRAGE) sequence was acquired on a 3T Siemens Tim-TRIO MR scanner with a 32-channel head coil (176 slices, slice thickness = 1 mm, TR = 2250ms, TE = 3.03ms, FoV = 256 × 256 mm^2^, flip angle = 9°, voxel resolution = 1 × 1 × 1 mm^3^). For each subject, T1-weighted images were processed using the CAT12v8 SPM12 toolbox, release v1853 [21]. Thereby, preprocessing steps included (i) the initial registration and bias field correction, (ii) the derivation of tissue probability maps (TPMs) of gray matter, white matter, and cerebrospinal fluid, and (iii) spatial normalization to a standard template (derived from N = 555; age range: 10–80 years; IXI-database; http://www.brain-development.org) with the help of geodesic shooting and Gauss–Newton optimisation-based diffeomorphic registration [22]. GMV (in ml) for all areas of the Julich-Brain atlas [23] as well as the total intracranial volume (ICV) were extracted from the 3D high-resolution T1-weighted structural brain images (for a detailed description of the brain image processing, see [24]).

### ML framework

A ML approach was chosen to investigate whether sex-specific cognitive profiles may be better predicted from structural imaging features (GMV values for all areas of the Julich-Brain atlas) as compared to whole-sample cognitive profiles. To do so, we compared ML estimations from predictions of the 1) whole-sample cognitive components in a) all participants, b) males only and c) females only, and 2) sex-specific cognitive components with a) a male component solution in males and b) a female component solution in females only.

From a methodological point, we aimed to ascertain that results are not specific to algorithm choice. Therefore, we compared three different prediction algorithms, i.e. Linear Support Vector Regression (linSVR), Elastic Net (EN) Regression, and Random Forest (RF) regression, which are commonly used in neuroimaging studies [10, 25, 26]. A repeated nested 10-fold cross-validation (CV) (10 repeats) was used to assess ML model performance. To additionally avoid data leakage, all hyperparameters were adjusted in the inner folds (5-fold CV). The following hyperparameters were optimized: regularization parameter C for linSVR (10^–4^ to 10^1^, 10 steps, logarithmic scale), the regularization parameters $\lambda$, and $\alpha$, for EN ($\lambda$: 10^–1^ to 10^2^, 10 steps, logarithmic scale; $\alpha$: 0.1 to 1, 10 steps), and the number of trees and tree depth for RF (number of trees: 100 or 1000; tree depth: 4, 6, 8, 10, 20, 40, None) [10]. Prediction performance was primarily assessed with the coefficient of determination (R^2^). Additionally, the mean absolute error (MAE) and Pearson's correlation (r) between true and predicted targets were calculated and are reported in the Suppl. Tables S1-6. The scikit-learn library (version: 0.22.1) in Python was used for all ML analyses ([27] https://scikit-learn.org/stable/).

#### Confounder analyses

Furthermore, to ensure that our predictions were not driven by potential confounders, we additionally assessed the impact of both, demographic variables (age and education; as assessed by the International Classification of Education, ISCED [28]) as well as ICV on the prediction. To assess the impact of demographic variables, age, and education were used as extra features to our ML models [10, 29]. Performance estimations were thus obtained for models based on (i) GMV, (ii) demographic variables (DV), and (iii) demographic variables and brain structural features (GMV + DV). Additionally, prediction performance for all three models (i.e. models [i], [ii], and [iii]) was compared between conditions without (no-deconf. condition) and with confound regression for the influence of intracranial volume (ICV) (deconf. condition) (based on the set up in [10, 30]).

#### Feature Importance

To discern relevant features for prediction, mean coefficients/importances were calculated for each ML model in each sample. Feature importance analyses were carried out in models without and with demographic features (DV) to gain a better understanding of the influence of age and education on prediction performance. Most important features were identified by (1) selecting the 25% of features with the highest coefficients/importances for each target in each algorithm (i.e. linSVR, EN, RF) and condition (no deconf. vs deconf.), and (2) choosing only those features represented in all algorithms and conditions. Afterwards, important features were plotted on the brain using Freeview implemented in FreeSurfer (https://surfer.nmr.mgh.harvard.edu/fswiki/FreeviewGuide).

#### ML validation analyses

Two supplementary analyses were performed to validate our ML pipeline. We decided on age and sex prediction as prior literature has shown robust prediction results for both from brain structural features [31]. In the current study, age was predicted using all three algorithms from the main analysis in the no-deconf. and deconf. condition. For sex prediction, a classification setup was chosen using a linear Support Vector Classifier (linSVC), Logistic Regression (Log), and Random Forest (RF) Classifier.

#### ML model comparisons

For contextualization of absolute prediction accuracies, ML model estimations were compared to those of a reference model, i.e. Dummy Regressor [30, 32]. The Dummy Regressor follows a simple prediction strategy of predicting the mean of the targets in the training set and serves as a baseline to which real model performance can be compared [27]. Specifically, the percentage of folds (folds > ref.), in which the real models outperform the dummy models, were calculated to provide a better basis for the interpretation of results.

## Results

### Prediction results for whole-sample cognitive profile

Initially, ML was used to assess the prediction power of brain structure, i.e., region-wise GMV within all parts of the Juelich Brain atlas [23], for cognitive profiles in older adults. Thereby, we made use of previously extracted cognitive profiles within age and education-matched groups of older males and females[16]. Importantly, three different component solutions were used in the current study derived from 1) a mixed group of males and females, i.e. whole-sample (W), 2) a group of females (F) only, and 3) a group of males (M) only (see Fig. 1A-D; [16]). ML estimations from predicting the whole-sample cognitive solution within the whole sample revealed satisfactory prediction performance for the first (W1 = [non-]verbal heterogeneous functions; mean R^2^ range = 0.07-0.12; in 82–94% of folds R^2^ > dummy regressor; see Table 1, Fig. 1E & Suppl. Tables S1), and decreased prediction performance for the second (W2 = [non-]verbal fluency and memory functions; mean R^2^ range = 0.02 to 0.06; in 68–88% of folds R^2^ > dummy regressor; see Table 1, Fig. 1E & Suppl. Tables S1) and third component (W3 = verbal short and working memory; mean R^2^ range = 0.00 to 0.03; in 55–77% of folds R^2^ > dummy regressor; see Table 1, Fig. 1E & Suppl. Tables S1) across algorithms and deconfounding conditions (no-deconf. and deconf.). Subsequently, the whole sample components were used to predict cognitive performance either in the male or female subsample, which revealed a slight ML performance decrease in females (W1: mean R^2^ range = 0.05-0.11; in 76–87% of folds R^2^ > dummy regressor; W2: mean R^2^ range = 0.02-0.05; in 64–79% of folds R^2^ > dummy regressor; W3: mean R^2^ range = -0.00-0.02; in 63–71% of folds R^2^ > dummy regressor; see Table 1, Fig. 1E & Suppl. Tables S1) and a substantial performance decrease in males (W1: mean R^2^ range = 0.05-0.07; in 73–82% of folds R^2^ > dummy regressor; W2: mean R^2^ range = 0.01-0.04; in 66–80% of folds R^2^ > dummy regressor; W3: mean R^2^ range = -0.04-0.00; in 50–62% of folds R^2^ > dummy regressor; see Table 1, Fig. 1E & Suppl. Tables S1) for all cognitive components across analytic choices. The general pattern of differences between components, however, remained in male and female subsamples (see Table 1, Fig. 1E & Suppl. Tables S1). Thus, it appeared that prediction performance differed not only between the three samples but also between the different general cognitive components.

**Table 1:** Prediction performance (R^2^) of whole-sample cognitive profile (W1-3) from brain structural features (GMV), brain structural features & demographic variables, i.e. age and education (GMV + DV), and demographic variables (DV) in the whole sample (W), female (F) and male (M) subsample

			GMV			GMV + DV			DV		
Sample	Target	Condition	EN	RF	linSVR	EN	RF	linSVR	EN	RF	linSVR
Whole sample	W1	No-deconf.	0.12 (0.08)	0.12 (0.09)	0.11 (0.09)	0.28 (0.09)	0.22 (0.09)	0.18 (0.10)	0.27 (0.09)	0.24 (0.10)	0.27 (0.10)
		Deconf.	0.08 (0.06)	0.09 (0.07)	0.07 (0.07)	0.25 (0.08)	0.20 (0.09)	0.16 (0.08)	0.26 (0.08)	0.24 (0.10)	0.26 (0.09)
	W2	No-deconf.	0.05 (0.07)	0.04 (0.07)	0.02 (0.08)	0.24 (0.08)	0.21 (0.09)	0.13 (0.09)	0.24 (0.08)	0.22 (0.11)	0.23 (0.10)
		Deconf.	0.06 (0.06)	0.06 (0.05)	0.03 (0.06)	0.24 (0.07)	0.22 (0.08)	0.14 (0.08)	0.24 (0.08)	0.22 (0.10)	0.24 (0.09)
	W3	No-deconf.	0.03 (0.05)	0.01 (0.07)	0.02 (0.06)	0.12 (0.07)	0.08 (0.07)	0.04 (0.08)	0.13 (0.08)	0.11 (0.09)	0.12 (0.08)
		Deconf.	0.01 (0.04)	0.01 (0.05)	0.00 (0.03)	0.12 (0.06)	0.08 (0.06)	0.03 (0.06)	0.14 (0.07)	0.11 (0.08)	0.11 (0.07)
Male subsample	W1	No-deconf.	0.07 (0.12)	0.06 (0.13)	0.06 (0.14)	0.22 (0.12)	0.13 (0.14)	0.10 (0.17)	0.24 (0.12)	0.17 (0.15)	0.23 (0.14)
		Deconf.	0.06 (0.09)	0.07 (0.09)	0.05 (0.12)	0.22 (0.10)	0.14 (0.12)	0.10 (0.14)	0.25 (0.11)	0.19 (0.13)	0.24 (0.13)
	W2	No-deconf.	0.01 (0.11)	0.01 (0.12)	0.01 (0.13)	0.14 (0.11)	0.12 (0.12)	0.04 (0.12)	0.17 (0.13)	0.10 (0.17)	0.16 (0.16)
		Deconf.	0.04 (0.07)	0.03 (0.09)	0.04 (0.08)	0.18 (0.09)	0.14 (0.09)	0.05 (0.10)	0.20 (0.11)	0.14 (0.14)	0.19 (0.14)
	W3	No-deconf.	-0.02 (0.09)	-0.04 (0.11)	-0.04 (0.13)	0.02 (0.10)	-0.02 (0.12)	-0.03 (0.14)	0.06 (0.11)	0.02 (0.12)	0.04 (0.14)
		Deconf.	0.00 (0.05)	-0.01 (0.07)	-0.02 (0.05)	0.06 (0.07)	0.01 (0.08)	-0.01 (0.07)	0.09 (0.09)	0.05 (0.11)	0.06 (0.10)
Female subsample	W1	No-deconf.	0.10 (0.11)	0.11 (0.13)	0.10 (0.11)	0.26 (0.12)	0.20 (0.12)	0.12 (0.13)	0.26 (0.12)	0.24 (0.15)	0.24 (0.12)
		Deconf.	0.05 (0.07)	0.06 (0.09)	0.05 (0.06)	0.22 (0.09)	0.16 (0.10)	0.08 (0.08)	0.25 (0.10)	0.21 (0.13)	0.24 (0.10)
	W2	No-deconf.	0.04 (0.12)	0.05 (0.12)	0.04 (0.12)	0.27 (0.12)	0.23 (0.13)	0.11 (0.14)	0.26 (0.13)	0.23 (0.15)	0.26 (0.15)
		Deconf.	0.04 (0.08)	0.04 (0.07)	0.02 (0.05)	0.26 (0.10)	0.20 (0.12)	0.08 (0.09)	0.26 (0.12)	0.22 (0.13)	0.25 (0.14)
	W3	No-deconf.	0.00 (0.10)	0.02 (0.13)	-0.00 (0.11)	0.15 (0.10)	0.11 (0.13)	0.02 (0.11)	0.15 (0.11)	0.11 (0.16)	0.15 (0.11)
		Deconf.	0.01 (0.05)	0.01 (0.09)	0.00 (0.04)	0.15 (0.07)	0.10 (0.09)	0.01 (0.07)	0.16 (0.08)	0.12 (0.13)	0.15 (0.08)

*Note: *Standard deviation (SD) appears in parentheses

### Prediction results for sex-specific cognitive profiles

Afterwards, sex-specific cognitive profiles (derived from either males or females) were predicted in the respective sex groups (see Table 2, Fig. 1F & Suppl. Tables S4). Differences in the predictability of cognitive solutions from GMV emerged for males and females. The male cognitive profile (M1: heterogenous functions; M2: visual WM & executive functions; M3: verbal WM and executive functions) could only be predicted to a limited degree. Region-wise GMV could not explain more than 8% variance across the different cognitive components (M1: mean R^2^ range = 0.04–08; in 74–84% of folds R^2^ > dummy regressor; M2: mean R^2^ range = 0.03-0.05, in 66–76% of folds R^2^ > dummy regressor; M3: mean R^2^ range = 0.03-0.06; in 72–80% of folds R^2^ > dummy regressor; see Table 2, Fig. 1F & Suppl. Tables S4). Moreover, no substantial predictability differences were observed for the different algorithms and deconfounding strategies.

**Table 2:** Prediction performance (R^2^) of sex-specific cognitive profiles (Males: M1-3; Females: F1-4) from brain structural features (GMV), brain structural features & demographic variables, i.e. age and education (GMV + DV), and demographic variables (DV) in female (F) and male (M) subsample

			GMV			GMV + DV			DV		
Sample	Target	Condition	EN	RF	linSVR	EN	RF	linSVR	EN	RF	linSVR
Male subsample	M1	No-deconf.	0.06 (0.12)	0.05 (0.14)	0.04 (0.13)	0.23 (0.12)	0.19 (0.14)	0.10 (0.14)	0.26 (0.14)	0.20 (0.17)	0.25 (0.16)
		Deconf.	0.08 (0.08)	0.07 (0.10)	0.05 (0.09)	0.26 (0.10)	0.21 (0.12)	0.12 (0.11)	0.28 (0.12)	0.23 (0.14)	0.27 (0.14)
	M2	No-deconf.	0.05 (0.11)	0.03 (0.13)	0.03 (0.14)	0.17 (0.11)	0.09 (0.13)	0.08 (0.14)	0.19 (0.11)	0.14 (0.15)	0.19 (0.13)
		Deconf.	0.05 (0.07)	0.04 (0.09)	0.04 (0.11)	0.18 (0.09)	0.10 (0.11)	0.08 (0.13)	0.21 (0.10)	0.16 (0.13)	0.20 (0.11)
	M3	No-deconf.	0.04 (0.12)	0.03 (0.14)	0.03 (0.12)	0.19 (0.11)	0.10 (0.14)	0.09 (0.14)	0.22 (0.12)	0.17 (0.15)	0.21 (0.14)
		Deconf.	0.06 (0.07)	0.06 (0.10)	0.04 (0.08)	0.20 (0.09)	0.14 (0.11)	0.10 (0.12)	0.24 (0.11)	0.19 (0.12)	0.23 (0.12)
Female subsample	F1	No-deconf.	0.11 (0.11)	0.12 (0.13)	0.10 (0.11)	0.29 (0.12)	0.22 (0.13)	0.14 (0.13)	0.29 (0.13)	0.26 (0.16)	0.28 (0.13)
		Deconf.	0.06 (0.07)	0.07 (0.09)	0.06 (0.07)	0.25 (0.10)	0.18 (0.10)	0.09 (0.09)	0.27 (0.11)	0.24 (0.14)	0.26 (0.11)
	F2	No-deconf.	0.03 (0.10)	0.02 (0.12)	0.02 (0.12)	0.21 (0.12)	0.19 (0.13)	0.08 (0.12)	0.21 (0.13)	0.18 (0.15)	0.20 (0.15)
		Deconf.	0.03 (0.07)	0.03 (0.08)	0.01 (0.05)	0.21 (0.10)	0.18 (0.12)	0.07 (0.09)	0.21 (0.11)	0.17 (0.13)	0.21 (0.13)
	F3	No-deconf.	0.11 (0.11)	0.12 (0.13)	0.11 (0.10)	0.29 (0.11)	0.21 (0.13)	0.14 (0.10)	0.28 (0.12)	0.22 (0.17)	0.26 (0.12)
		Deconf.	0.07 (0.07)	0.09 (0.10)	0.06 (0.06)	0.25 (0.09)	0.18 (0.12)	0.09 (0.07)	0.26 (0.10)	0.21 (0.15)	0.25 (0.11)
	F4	No-deconf.	0.02 (0.11)	0.05 (0.14)	0.04 (0.11)	0.23 (0.11)	0.21 (0.13)	0.10 (0.13)	0.24 (0.12)	0.22 (0.17)	0.24 (0.13)
		Deconf.	0.02 (0.06)	0.03 (0.08)	0.01 (0.05)	0.22 (0.09)	0.19 (0.11)	0.08 (0.08)	0.24 (0.10)	0.20 (0.14)	0.23 (0.10)

*Note: *Standard deviation (SD) appears in parentheses

The female cognitive profile displayed a mixed pattern of predictability from region-wise GMV. The female cognitive components related to visual short-term and working memory and executive functions (F1: mean R^2^ range = 0.06-0.12; in 79–89% of folds R^2^ > dummy regressor; F3: mean R^2^ range = 0.06-0.11; in 79–91% of folds R^2^ > dummy regressor; see Table 2, Fig. 1F & Suppl. Tables S4) could be better predicted than those related to (non-)verbal fluency and verbal short-term and working memory (F2: mean R^2^ range = 0.01-0.03; in 58–75% of folds R^2^ > dummy regressor; F4: mean R^2^ range = 0.01-0.05; in 63–73% of folds R^2^ > dummy regressor; see Table 2, Fig. 1F & Suppl. Tables S4). As such, up to 11 to 12% of the variance could be explained in components F1 and F3, while only between 1 to 5% of the variance could be explained in components F2 and F4 from the input data. Results appeared similar across different analytic choices.

Despite the emergence of predictability differences for sex-specific cognitive profiles, it should be emphasized that explained variances remained relatively small for all components and that described effects were only very modest in size (see Fig. 1E & F). Nevertheless, addressing sex-specific cognitive profiles allowed a more fine-grained investigation of predictability, which may have not been possible by simply investigating the whole-sample solution.

### The impact of age and education on cognitive prediction results

**Fig. 2:**
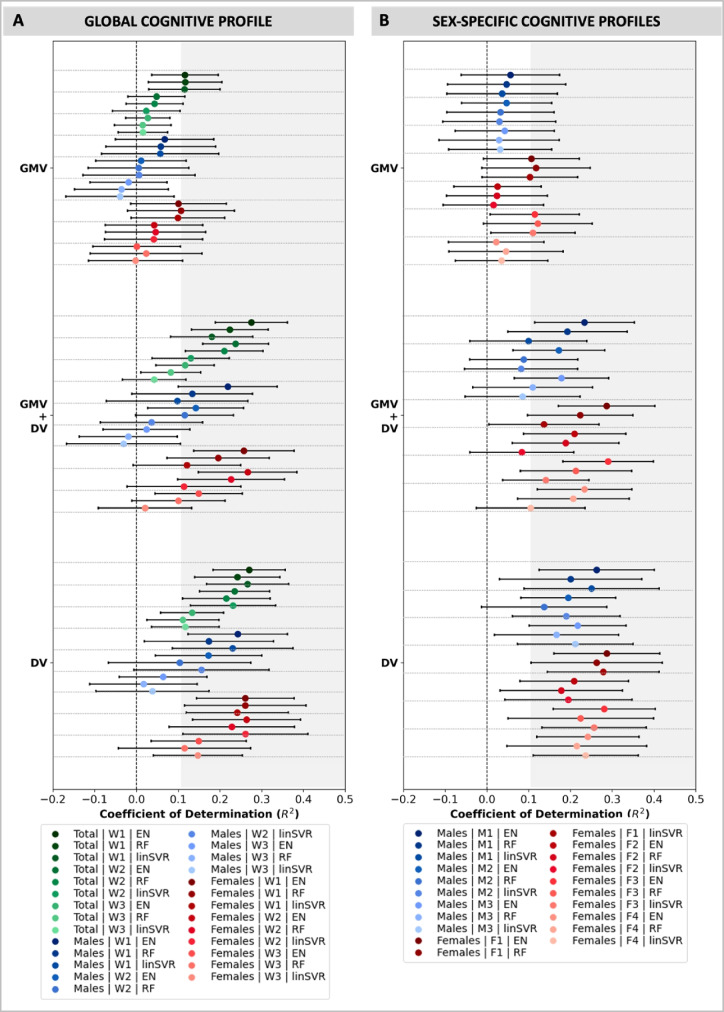
Prediction performance of whole-sample (**A**) and sex-specific cognitive profiles (**B**) from (i) region-wise GMV, (ii) regions-wise GMV and demographic variables, i.e. age and education (GMV + DV), (iii) demographic variables, i.e. age and education (DV). ML estimations are displayed for the global cognitive profile (left) in the whole sample, in females and males as well as for sex-specific profiles (right) in males and females across different algorithms, i.e. Elastic Net (EN) regression, Random Forest (RF) regression and Linear Support Vector Regression (linSVR). The coefficient of determination (R^2^) is displayed with error bars representing the standard deviation (SD). The grey shaded areas indicate R^2^ > 0.1 suggesting satisfactory prediction performance. Results from no-deconf. condition shown

In a second step, we examined the predictability of cognitive profiles (whole-sample & sex-specific) in older adults from demographic variables, i.e. age and education, only and in conjunction with structural imaging features, i.e., region-wise GMV within all parts of the Juelich Brain atlas [23]. Results were, then, compared to those from using only brain structural features. This was done to get a more detailed understanding of the influence of age and education on prediction performance. Since previous work showed that demographic factors (among others) might be successful in predicting cognitive performance [25], we assessed whether the prediction performance would benefit from including the two in the ML approach. Results showed the lowest prediction performance for models solely derived from regional brain structural features, i.e. GMV (see Fig. 2). In contrast, the inclusion of age and education in the ML models resulted in a remarkable increase in prediction performance (see Table 1–2, Fig. 2 and Suppl. Tables S1-6). For example, while models based on GMV could explain only between 7 and 12% of variance in component one (W1) of the global component solution, models including extra features, i.e. age and education, could explain between 16 and 28% in variance in W1 across algorithms and deconfounding conditions (DV: mean R^2^ range = 0.24-0.27; in 99–100% of folds R^2^ > dummy regressor; GMV + DV: mean R^2^ range = 0.16-0.28; in 96–100% of folds R^2^ > dummy regressor, see Table 1, Fig. 2A & Suppl. Tables S2-3). Thus, explained variance increases of up to nearly 20% were observed, once age and education were added to ML models. Interestingly, these remarkable increases in prediction performance were not present for all components. Thus, for the global cognitive profile, stronger positive effects of the inclusion of age and education on prediction performance were found for component one (W1 = [non-]verbal heterogenous functions; mean R^2^ range = 0.16-0.28; in 96–100% of folds R^2^ > dummy regressor) and two (W2 = [non-]verbal fluency and memory functions; mean R^2^ range = 0.13-0.24; in 92–100% of folds R^2^ > dummy regressor) compared to component three (W3 = verbal short and working memory; mean R^2^ range = 0.03-0.14; in 76–99% of folds R^2^ > dummy regressor) (see Table 1, Fig. 2A & Suppl. Tables S2-3). A similar pattern was found for the global components applied to either males or females (see Table 1, Fig. 2A & Suppl. Tables S2-3). Overall, component three (W3) was found to be difficult to predict from both brain structure and the demographic variables age and education in all three different samples across analytic options.

Turning to the sex-specific components (derived from either males or females), the inclusion of age and education in ML models equally led to increases in prediction performance. While low prediction performance was observed for the male component solution based on brain structural data (M1-3: mean R^2^ range = 0.03-0.08; in 76–84% of folds R^2^ > dummy regressor; see Table 2, Fig. 2B & Suppl. Tables S4), satisfactory prediction performance was encountered once models were based on (DV) or included (GMV + DV) age and education (M1-3: mean R^2^ range = 0.08-0.28; in 79–100% of folds R^2^ > dummy regressor; see Table 2, Fig. 2B & Suppl. Tables S5-6). Results tended to be quite similar for different components for each algorithm across deconfounding strategies with a slight advantage of adding age and education to component one (M1 = [non-]verbal heterogeneous functions) (see Table 2, Fig. 2B & Suppl. Tables 5–6). In females, adding age and education also increased prediction performance. Nevertheless, predictability differences between cognitive components were preserved, when adding the confounding variables (F1 & F3: mean R^2^ = 0.09-0.29; in 85–100% of folds R^2^ > dummy regressor; F2 & F4: mean R^2^ = 0.07-0.23; in 79–99% of folds R^2^ > dummy regressor; see Table 2, Fig. 2B & Suppl. Tables 5–6). Overall, adding age and education substantially boosted prediction power across models and samples.

## Feature importance

**Fig. 3:**
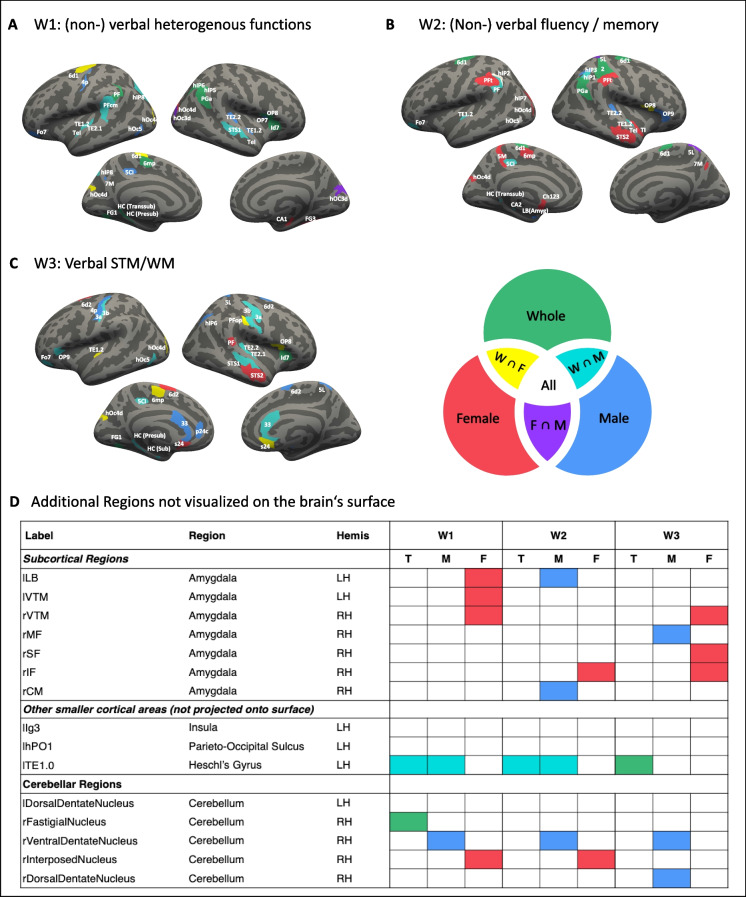
Important features from the whole-sample cognitive component solution (W1-3) prediction plotted on a standard brain’s surface reconstruction. Hemis = hemisphere; LH = left hemisphere; RH = right hemisphere; T = whole sample, M = males, F = females

Finally, we explored the feature importance for the prediction of cognitive profiles in both the global as well as the sex-specific cognitive profiles. Figure 3 represents the top 25% features that are important to predict the global cognitive profile in the whole group as well as males and females separately (consisting of the different ML algorithms: EN, RF, linSVR). Concerning the whole group component solution, widespread networks seem to be related to the prediction of cognitive profiles. Focusing on the whole group, the first cognitive component W1 ((non-) verbal heterogeneous functions) seems to be best predictable from features within the motor system (left cytoarchitectonic areas 6d1 and 6mp), the inferior parietal lobule (e.g. left areas PF, PFcm and right PGa), the superior temporal gyrus (i.e. right area STS1) together with the Heschl’s gyrus (e.g. left areas TE1.2 and TE2.1) and the extrastriate cortex (areas left hOc4d) (see Fig. 3A). Importantly, when it comes to the prediction of the global component solution in either males or females, the relevant features for prediction do not show much overlap in the two sexes (see Fig. 3, the overlap is indicated in purple). Generally, important features in the male group show more overlap with the whole group, with the most important features being located within the temporal (i.e. superior temporal sulcus and gyrus and Heschl’s gyrus) and parietal lobule. In contrast to that, within females, the most important features are located within the visual system, the amygdala, and the hippocampus. The second component W2 ((Non-)verbal fluency / memory) reveals a similar picture. Males and females show quite distinct relevant features for prediction throughout the brain. While all, i.e. the whole group, males and females, show most of the relevant features in the inferior and superior parietal lobules, males and females refer to different proportions of these with only few commonalities (see Fig. 3B). Regarding the third component W3 (Verbal STM/WM), the primary somatosensory cortex gains in importance, in the whole as well as male group (areas 3a, 3b), but not in females. In addition, the anterior cingulate cortex appeared important in both males and females for the prediction of W3, but again different portions (males: bilateral area 33 and left area p24c, females: bilateral area s24) (see Fig. 3C).

The assessment of important features for the prediction of the sex-specific component solutions in males and females further supported the observation from the whole group's global cognitive profile: different parts of the brain appear to be important for the sex-specific cognitive component prediction.

**Fig. 4:**
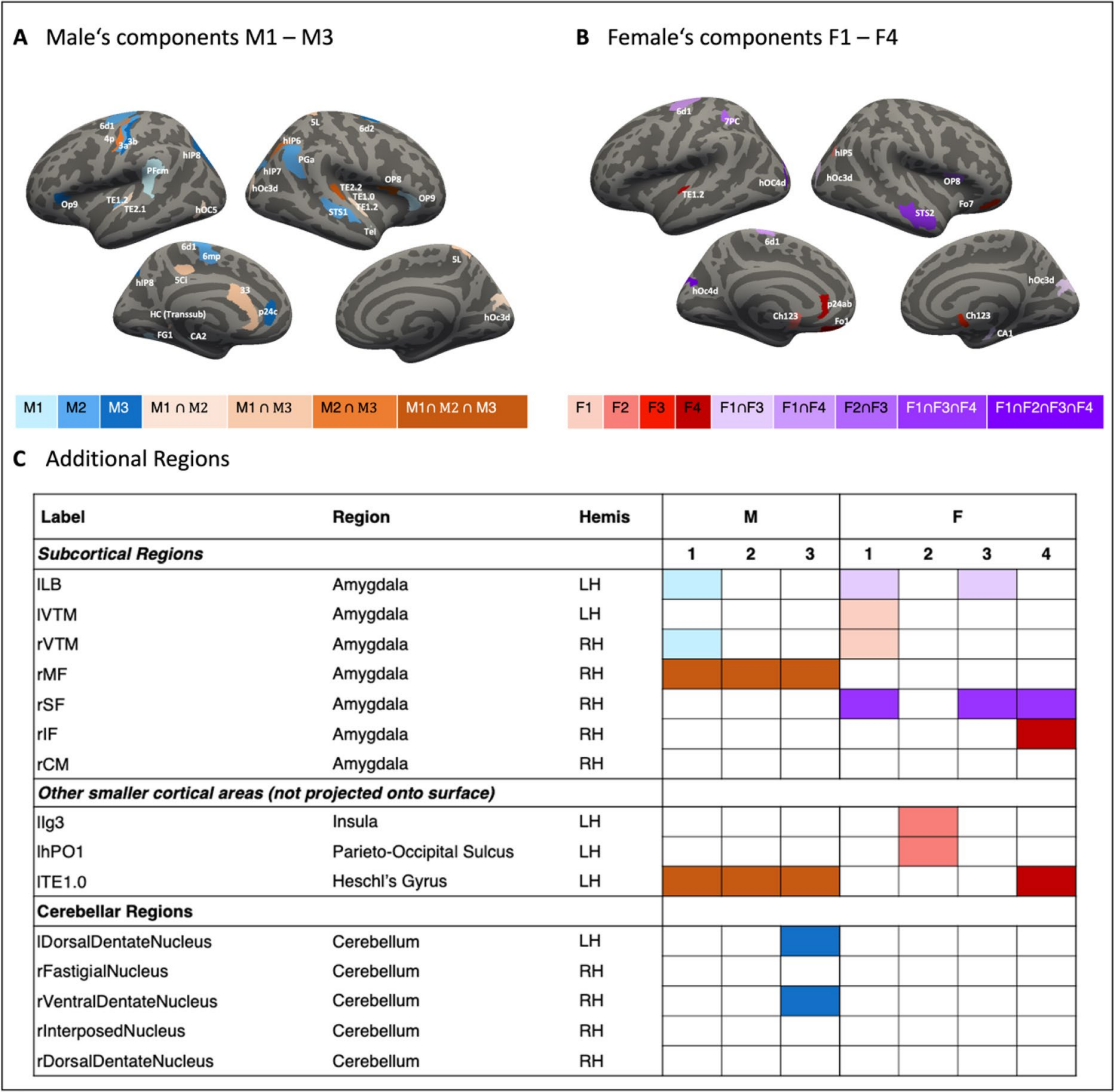
Important features from the male and female cognitive component solutions (M1-3; F1-4) prediction plotted on a standard brain’s surface reconstruction. Hemis = hemisphere; LH = left hemisphere; RH = right hemisphere

In males, for example, a higher amount of important features was found for predicting cognitive profiles. All cognitive components relied, e.g. on the superior temporal and Heschl gyrus (areas TE1.0, TE2.2), and the frontal operculum (area OP8). In addition, the highest portion of important features was found within the temporal lobe for components M1 and M2 (e.g. superior temporal gyrus), while component M3 showed the highest number of important features within the inferior parietal lobe (see Fig. 4A).

For females, on the other hand, a smaller number of features, i.e. GMV regions, seem to be important for predicting cognitive profiles. For example, the extrastriate cortex, i.e. left hOc4d region, appeared to be relevant for the prediction of all cognitive components. Furthermore, the amygdala seemed to be important in the prediction of component F1, while features within the motor system and occipital lobe were more relevant for component F2. Components F3 and F4, in turn, were associated with widespread brain regions (see Fig. 4B).

### Validation analyses

To validate our ML approach, we performed, further, age and sex predictions. Across analytic choices, we observed robust prediction results for both age (mean R^2^ range = 0.26-0.39; in 99–100% of folds R^2^ > dummy regressor, see Suppl. Tables S7 & Suppl. Figure S8) and sex (mean accuracy range = 76–81%; in 100% of folds R^2^ > dummy regressor, see Suppl. Tables S9 & Suppl. Figure S10) from brain structural data, in line with previous literature [31, 33].

## Discussion

The current study aimed to examine whether the predictability of cognitive performance from brain structure differs between general cognitive profiles derived from the whole sample including both sexes and sex-specific cognitive profiles based on brain structural features i.e. region-wise GMV, in older adults from the 1000BRAINS study using an ML approach. Present findings generally showed that prediction performance of a whole-sample cognitive profile, i.e. including males and females in one group, from brain structure was limited in older adults across analytic choices (only one of the three components (W1, heterogeneous cognitive functions) reached an R^2^ > 0.1). Applying this component solution to males and females separately, further, showed that this global cognitive component solution did not exceed the results for the whole group. While prediction performance within the female subsample was at a similar level compared to the whole group, prediction accuracies in males were found to be even lower compared to the whole sample. Although subsequent investigations of sex-specific cognitive components overall were not able to surpass ML results from the whole-sample cognitive profile, they uncovered patterns of predictability differences that were not discernible in the whole-sample solution. Particularly, low predictability was observed for all cognitive components (M1-3) from the male solution, while predictability differences surfaced between different female cognitive components, i.e. visual short-term and working memory and executive functions vs (non-)verbal fluency, which were better predictable, and verbal short-term memory (F1 & 3 vs F2 & 4).

Differences between males and females in cognitive abilities have long attracted the attention of researchers. While earlier reports hinted at general cognitive performance differences, e.g. superior performance of males in visual tasks and of females in verbal tasks (e.g. [34, 35]), newer studies have suggested that males and females may rather differ in their cognitive processing style (global vs local) giving rise to distinct cognitive profiles [16, 36]. This finding, in turn, may be of interest in the prediction of cognitive functioning in older adults providing a potential explanation for the so far only restricted success in predicting cognition in healthy older adults [11, 30, 37]. Hence, our results emphasized that prediction accuracies for the whole sample using sex-specific cognitive profiles (highest mean R^2^: 0.12 [female] & 0.08 [male]) did not exceed those using a general cognitive profile (highest mean R^2^: 0.12), or in female (highest mean R^2^: 0.11) and male (highest mean R^2^: 0.07) subsamples. Thus, it appeared that sex differences in the optimal cognitive solution may not account for the low overall prediction accuracies previously encountered in studies focusing on older adults.

So far, it remains uncertain to what extent behavior or cognition can be predicted from brain imaging data [38, 39]. Recent research suggests that the prediction power of cognitive abilities from different imaging modalities in different samples, i.e. young, old, across the lifespan, is limited [10, 11, 29, 37, 40]. For example, Rasero, Sentis [29] showed that multimodal data did not explain more than 8% of the variance in global cognitive function in a large sample of younger adults (HCP S1200 release). Hilger, Winter [11] found that intelligence could only be predicted from GMV with high error rates and correlation values between true and predicted scores of up to r = 0.30 in a large sample across the lifespan. Further, findings from a middle-aged to older adult cohort (UK Biobank) demonstrated that prediction accuracies of fluid intelligence from sMRI data do not surpass R^2^ = 0.04 [37]. In a similar vein, in a large sample of older adults (1000BRAINS) best prediction accuracies of global cognition from multimodal data, i.e. region-wise GMV, functional connectivity (FC), and structural connectivity (SC) estimates, reached a maximum of 14% of the variance (R^2^) in absence of confounder control [30]. Current findings fall into the same range and extend it to the prediction of sex-specific cognitive profiles in a large sample of older adults. In this context, region-wise GMV did not explain more than 12% of the variance across the different cognitive targets, i.e. whole-sample and sex-specific cognitive profiles. In terms of the correlation between true and predicted scores, values were not found to be greater than r = 0.38 in the current study. Thus, it appeared that brain structure, GMV in particular, only captures a limited extent of cognitive performance differences in older age and that successful and reliable prediction of cognitive abilities from brain imaging data remains a challenging endeavor.

Even though we did not observe a prediction power advantage for sex-specific cognitive profiles when investigating the whole group, their use as cognitive targets uncovered previously unnoted patterns of prediction performance in males and females. Generally, higher predictability of cognitive profiles (sex-specific and whole-sample) was observed for females compared to male older adults. Prediction accuracies for all male cognitive components were similarly low (mean R^2^ range: 0.03–0.08). In contrast, a more divergent set of effects unraveled for females. In this context, visual short-term and working memory and executive functions (mean R^2^ range for F1 & 3: 0.06–0.12) could be better predicted than (non-)verbal fluency and verbal short-term memory (mean R^2^ range for F2 & 4: 0.01–0.05). Similar findings have been previously mainly reported in younger samples (but see also [18, 19]). For example, Jiang, Calhoun [41] have demonstrated that intelligence can be predicted more accurately from FC data in females (r = 0.72) compared to males (r = 0.46) across three different cohorts of young adults. Along the lines, Cui, Su [42] have shown that reading comprehension (measured by two different tests: picture vocabulary & oral reading recognition) could be predicted to a higher extent in females (r = 0.40–0.42) than males (r = 0.32–0.36) and comparably in females (r = 0.40–0.42) and the whole sample (r = 0.40–0.43) from GMV in a sample from the HCP. With the current study, we extend these prior findings to the prediction of sex-specific cognitive profiles from region-wise GMV in older adults (1000BRAINS), which to our knowledge has not been investigated before. Particularly, present results emphasize predictability differences of sex-specific cognitive profiles between older males and females as well as predictability differences among distinct sex-specific cognitive profiles, especially in older females. Thus, highlighting that differences in cognitive processing accompanied by differential predictability between males and females may also be preserved into higher ages and may not solely pertain to younger adults. Furthermore, findings add to initial reports of the feasibility and potential of using brain structural data in brain-behaviour relationship research beyond the more common use of functional connectivity data for prediction [43].

One potential explanation for these general differences in predictability between the sexes may be related to the neural underpinnings corresponding to the different processing styles (local vs. global) [16, 19, 44]. This is supported by the feature importance analysis in the current study. Across the cognitive components, a greater number of relevant features was found for males as compared to females, suggesting that regional involvement is less specific to a certain cognitive function and more representative of general cognitive processes, i.e. global processing. For instance, males showed the primary motor (e.g. area 4p), primary sensory (e.g. area 3a and 3b), and primary auditory (e.g. area TE1.2 and TE2.2) cortices to be relevant for the prediction of cognitive performance across cognitive functions. Alterations in these primary processing brain regions have been associated with cognitive decline in older adults [44, 45]. Thus, together with regions within, e.g. the inferior parietal lobule and the anterior cingulate cortex, these regions might be related to general cognitive functioning in older males, which is in line with the more global cognitive profile seen in males. As such, lower predictability of cognitive abilities in males may be related to a more integrative network of different brain regions, which may finally result in weak correspondence between the GMV of a particular region and the cognitive target [19, 44].

In contrast, female cognitive components were differentially related to brain structural patterns of important features, with less involvement of the primary processing cortices. Strikingly, fewer features seem to be important in the prediction of cognitive profiles. Here, we, for instance, observed an involvement of the amygdala in the first component F1 (visual working memory), which has recently been shown to be important in the regulation of attentionally demanding tasks, i.e. working memory [46]. Region Fo7 within the lateral orbitofrontal cortex, which is related to covert reading abilities, represents an important feature for predicting the fourth cognitive component, verbal working memory [47]. The current results, hence, emphasized more specialized processing of information in females, i.e. local processing, that may allow for an easier mapping between brain and cognition. Thus, it can be argued that predictability differences between males and females might be driven by the different processing styles at the brain level, which may become particularly evident when examining sex-specific cognitive profiles. As such, advocating for the examination of not only general cognitive profiles but also sex-specific cognitive profiles. This might be particularly relevant not only in higher ages in the search for a biomarker for age-related cognitive decline but also in clinical samples on the road to precision medicine. Especially, in light of differences in the prevalence of pathological conditions accompanied by cognitive decline, e.g. Alzheimer's disease and Parkinson's disease, between males and females, it might become essential to consider sex-specific cognitive profiles not only in diagnosis and biomarker research but also for individualized treatment opportunities [14, 15, 48, 49].

It needs to be emphasized that some cognitive abilities, e.g. executive and memory functions, are more strongly impacted by the aging process and tend to decline more strongly than others, e.g. language functions [5, 50]. These differences may also be expressed in different extents of predictability. As such, it appears advisable to also compare prediction performance between the different cognitive components for each solution, i.e. whole-sample and sex-specific. Across all solutions and analytic options, components related to verbal cognitive functions appeared to be predicted worse compared to all other components, i.e. heterogeneous functions, visual short-term and working memory, and executive functions. While results stand in contrast to findings in younger cohorts potentially due to differences in cognitive functions further aggravating only during the aging process (e.g. [18, 29]), they fit previous accounts in studies across the lifespan and older cohorts [10, 51, 52]. For instance, Feng, Wang [52] revealed that language functions could be predicted to a considerably smaller degree than attention and executive functions from SC data in two large older cohorts, i.e. HCP-A and BARBI. Along these lines, prior results from our group further substantiate this notion. Across analytic options, language functions resulted in the lowest prediction results from multimodal data in a large sample of older adults from 1000BRAINS [30]. This pattern of results has been replicated in the current study and was equally found for male and female subsamples and sex-specific cognitive profiles. As such, it appears that language functions may not only follow different aging trajectories [5, 50] but also differ in their predictability. A potential explanation for these results may relate to the relative stability of language functions in aging potentially resulting in a lower variability as well as the influence of other factors such as education that may explain a substantial amount of variance in the target [53, 54]. Hence, current results support the notion that not all cognitive functions may be predicted equally well in older age and that particular predictability of language functions appears restricted.

Demographic variables, e.g. age and education, have been found to exert a substantial influence on ML performance [55]. Thus, we performed additional confounder analyses in the current study by comparing brain-based models to those including demographic factors as extra features (GMV + DV) or only features (DV) [29, 30]. Across cognitive targets and analytic options, the use of age and education as input features to ML led to drastic increases in prediction performance. As such, increases of up to 20% in explained variance in cognitive components could be observed. In this context, it should be noted that differences in ML performance between components and subsamples were largely preserved in models with demographic variables. The ML performance boost linked to including demographic variables is in line with prior literature [29, 30, 37, 56, 57]. For instance, Rasero, Sentis [29] showed that the inclusion of age, sex, and education in models led to performance increases of up to 10% in explained variance (R^2^) in global cognition in younger adults. In older adults, similar increases in prediction performance have been reported [30, 57]. In the present study, brain structure does not appear to add extra information to the prediction of cognitive abilities beyond demographic variables, similar to findings in [10, 25]. Particularly, current findings suggest that even after accounting for potential sex differences, the influence of age and education on ML performance persists. Thus, it appears that age and education may account for a substantial amount of variance overshadowing brain-behaviour relationships, even in already sex-stratified analyses, in the current sample of older adults from the 1000BRAINS study.

## Limitations

In the current study, we focused on the prediction of general and sex-specific cognitive profiles from brain structural data. Overall, results suggested limited prediction power of region-wise GMV for cognition prediction in older age. In future studies, it might, therefore, become indispensable to try other structural features and input modalities, e.g. dynamic functional connectivity or task-based functional imaging data [25, 58, 59], multimodal approaches, e.g. brain structure and function [29, 30], and other non-brain data, e.g. lifestyle information, genetic information and health information (i.e. hormonal status) [37], to achieve satisfactory ML performance in the future [60].

Furthermore, it should be kept in mind that sex in the current study was considered to be binary with all females and males displaying the respective sex-specific cognitive profiles. Recent views of a more continuum-like or differential expression of male and female characteristics representation of sex, thus, fall short of being fully appreciated in the present study [61]. Thus, it would be interesting to investigate in future studies how strongly individuals express the respective cognitive profile, whether it corresponds to their biological sex, and whether this results in predictability differences. This would potentially allow taking a more individualistic perspective of cognitive profiles that may ultimately boost prediction performance.

So far, sex-specific cognitive profiles have to our knowledge not been predicted before in older adults. Current results, thus, provide new insights into the prediction power of the whole sample and sex-specific cognitive profiles from brain structure in older adults from the 1000BRAINS study. In future studies, it would be interesting to investigate if sex-specific cognitive profiles and prediction results replicate in other larger cohorts of older adults and patterns of predictability differences can also be found in younger cohorts or whether these only pertain to older cohorts.

## Conclusion

The present study investigated the predictability of general and sex-specific cognitive profiles from brain structural data, i.e. region-wise GMV, in older adults from the 1000BRAINS study using ML. The investigation of sex-specific cognitive components uncovered new patterns of predictability differences beyond the whole-sample solution. At the same time, results also stressed the challenging nature of cognition prediction from imaging data in older adults.

### Supplementary Information

Below is the link to the electronic supplementary material.

Supplementary file1 (PDF 368 KB)

## Data Availability

Due to local regulations of data acquisition and usage, data of 1000BRAINS are available upon request from the responsible PI.

## References

[1] Oschwald J (2019). Brain structure and cognitive ability in healthy aging: a review on longitudinal correlated change. Rev Neurosci.

[2] Gorbach T (2017). Longitudinal association between hippocampus atrophy and episodic-memory decline. Neurobiol Aging.

[3] Hardcastle C (2020). Contributions of hippocampal volume to cognition in healthy older adults. Front Aging Neurosci.

[4] Kaup AR (2011). A review of the brain structure correlates of successful cognitive aging. J Neuropsychiatry Clin Neurosci.

[5] Salthouse TA (2010). Selective review of cognitive aging. J Int Neuropsychol Soc.

[6] Zhang T, et al. Predicting MCI to AD conversation using integrated sMRI and rs-fMRI: machine learning and graph theory approach. Front Aging Neurosci. 2021;13:688926. 10.3389/fnagi.2021.688926 PMC8375594 34421570

[7] Hojjati SH, Babajani-Feremi A, Alzheimer’s Disease Neuroimaging Initiative (2021). Prediction and modeling of neuropsychological scores in alzheimer's disease using multimodal neuroimaging data and artificial neural networks. Front Comput Neurosci.

[8] Nemali A, et al. Individualized gaussian process-based prediction of memory performance and biomarker status in ageing and Alzheimer’s disease. bioRxiv. 2022. 10.1016/j.media.2023.102913 37660483

[9] Moradi E (2017). Rey's auditory verbal learning test scores can be predicted from whole brain MRI in Alzheimer's disease. Neuroimage Clin.

[10] Krämer C, et al. Classification and prediction of cognitive performance differences in older age based on brain network patterns using a machine learning approach. Network Neurosci. 2023;7(1):122–147. 10.1162/netn_a_00275 PMC10270720 37339286

[11] Hilger K (2020). Predicting intelligence from brain gray matter volume. Brain Struct Funct.

[12] Maitland SB (2010). Gender differences and changes in cognitive abilities across the adult life span. Aging Neuropsychol Cogn.

[13] Laws KR, Irvine K, Gale TM (2016). Sex differences in cognitive impairment in Alzheimer's disease. World J Psychiatry.

[14] Cholerton B (2018). Sex differences in progression to mild cognitive impairment and dementia in Parkinson's disease. Parkinsonism Relat Disord.

[15] Sohn D (2018). Sex differences in cognitive decline in subjects with high likelihood of mild cognitive impairment due to Alzheimer's disease. Sci Rep.

[16] Jockwitz C (2021). Cognitive profiles in older males and females. Sci Rep.

[17] Caspers S (2014). Studying variability in human brain aging in a population-based German cohort-rationale and design of 1000BRAINS. Front Aging Neurosci.

[18] Dhamala E (2022). Shared functional connections within and between cortical networks predict cognitive abilities in adult males and females. Hum Brain Mapp.

[19] Jiang R (2020). Multimodal data revealed different neurobiological correlates of intelligence between males and females. Brain Imaging Behav.

[20] Schmermund A (2002). Assessment of clinically silent atherosclerotic disease and established and novel risk factors for predicting myocardial infarction and cardiac death in healthy middle-aged subjects: rationale and design of the Heinz Nixdorf RECALL Study. Risk factors, evaluation of coronary calcium and lifestyle. Am Heart J.

[21] Franke K, Gaser C (2019). Ten years of BrainAGE as a neuroimaging biomarker of brain aging: what insights have we gained?. Front Neurol.

[22] Ashburner J, Friston KJ (2011). Diffeomorphic registration using geodesic shooting and Gauss-Newton optimisation. Neuroimage.

[23] Amunts K (2020). Julich-Brain: A 3D probabilistic atlas of the human brain's cytoarchitecture. Science.

[24] Jockwitz C, et al. Characterization of the angular gyrus in an older adult population: a multimodal multilevel approach. Brain Struct Funct. 2023;228:83–102. 10.1007/s00429-022-02529-3 PMC9813183 35904594

[25] Hebling Vieira B (2022). Predicting future cognitive decline from non-brain and multimodal brain imaging data in healthy and pathological aging. Neurobiol Aging.

[26] Cui Z, Gong G (2018). The effect of machine learning regression algorithms and sample size on individualized behavioral prediction with functional connectivity features. Neuroimage.

[27] Pedregosa F (2011). Scikit-learn: machine learning in Python. J Mach Learn Res..

[28] Organisation for Economic Co-operation and Development. Classifying educational programmes: manual for ISCED-97 implementation in OECD countries. Paris: Organisation for Economic Co-operation and Development. 1999;113.

[29] Rasero J (2021). Integrating across neuroimaging modalities boosts prediction accuracy of cognitive ability. PLoS Comput Biol.

[30] Krämer C, et al. Prediction of cognitive performance differences in older age from multimodal neuroimaging data. Geroscience. 2023. 10.1007/s11357-023-00831-4 PMC10828156 37308769

[31] Weis S (2020). Sex classification by resting state brain connectivity. Cereb Cortex.

[32] Engemann DA, et al. Combining magnetoencephalography with magnetic resonance imaging enhances learning of surrogate-biomarkers. Elife. 2020;9:e54055. 10.7554/eLife.54055 PMC7308092 32423528

[33] Liem F (2017). Predicting brain-age from multimodal imaging data captures cognitive impairment. Neuroimage.

[34] Munro CA (2012). Sex differences in cognition in healthy elderly individuals. Neuropsychol Dev Cogn B Aging Neuropsychol Cogn.

[35] Weiss EM (2006). Sex differences in clustering and switching in verbal fluency tasks. J Int Neuropsychol Soc.

[36] Pletzer B, Scheuringer A, Scherndl T (2017). Global-local processing relates to spatial and verbal processing: implications for sex differences in cognition. Sci Rep.

[37] Dadi K, et al. Population modeling with machine learning can enhance measures of mental health. Gigascience. 2021;10(10):giab071. 10.1093/gigascience/giab071 PMC8559220 34651172

[38] Woo CW (2017). Building better biomarkers: brain models in translational neuroimaging. Nat Neurosci.

[39] Schulz M-A, et al. Performance reserves in brain-imaging-based phenotype prediction. bioRxiv. 2022. 10.1016/j.celrep.2023.113597 PMC11215805 38159275

[40] Boeke EA, Holmes AJ, Phelps EA (2020). Toward robust anxiety biomarkers: a machine learning approach in a large-scale sample. Biol Psychiatry Cogn Neurosci Neuroimaging.

[41] Jiang R (2020). Gender differences in connectome-based predictions of individualized intelligence quotient and sub-domain scores. Cereb Cortex.

[42] Cui Z (2018). Individualized prediction of reading comprehension ability using gray matter volume. Cereb Cortex.

[43] Genon S, Eickhoff SB, Kharabian S (2022). Linking interindividual variability in brain structure to behaviour. Nat Rev Neurosci.

[44] Stumme J (2020). Functional network reorganization in older adults: Graph-theoretical analyses of age, cognition and sex. Neuroimage.

[45] Perry A (2017). The independent influences of age and education on functional brain networks and cognition in healthy older adults. Hum Brain Mapp.

[46] de Voogd LD, Hermans EJ (2022). Meta-analytic evidence for downregulation of the amygdala during working memory maintenance. Hum Brain Mapp.

[47] Wojtasik M (2020). Cytoarchitectonic characterization and functional decoding of four new areas in the human lateral orbitofrontal cortex. Front Neuroanat.

[48] Ferretti MT, Galea LA (2018). Improving pharmacological treatment in brain and mental health disorders: the need for gender and sex analyses. Front Neuroendocrinol.

[49] Karstens AJ, Maynard TR, Tremont G. Sex-specific differences in neuropsychological profiles of mild cognitive impairment in a hospital-based clinical sample. J Int Neuropsychol Soc. 2023;1–10. 10.1017/S1355617723000085 36866579

[50] Hedden T, Gabrieli JD (2004). Insights into the ageing mind: a view from cognitive neuroscience. Nat Rev Neurosci.

[51] Tsapanou A, Stern Y, Habeck C (2020). Optimized prediction of cognition based on brain morphometry across the adult life span. Neurobiol Aging.

[52] Feng G (2022). Methodological evaluation of individual cognitive prediction based on the brain white matter structural connectome. Hum Brain Mapp.

[53] Jockwitz C (2019). Generalizing age effects on brain structure and cognition: a two-study comparison approach. Hum Brain Mapp.

[54] Opdebeeck C, Martyr A, Clare L (2016). Cognitive reserve and cognitive function in healthy older people: a meta-analysis. Neuropsychol Dev Cogn B Aging Neuropsychol Cogn.

[55] Snoek L, Miletic S, Scholte HS (2019). How to control for confounds in decoding analyses of neuroimaging data. Neuroimage.

[56] Yu J (2020). The individualized prediction of cognitive test scores in mild cognitive impairment using structural and functional connectivity features. Neuroimage.

[57] Yeung HW, et al. Predicting sex, age, general cognition and mental health with machine learning on brain structural connectomes. Hum Brain Mapp. 2023;44:1913–1933. 10.1002/hbm.26182 PMC9980898 36541441

[58] Fong AHC (2019). Dynamic functional connectivity during task performance and rest predicts individual differences in attention across studies. Neuroimage.

[59] Sripada C (2020). Toward a "treadmill test" for cognition: Improved prediction of general cognitive ability from the task activated brain. Hum Brain Mapp.

[60] Murdaca G, et al. Vitamin D and folate as predictors of MMSE in Alzheimer’s disease: a machine learning analysis. Diagnostics (Basel). 2021;11(6):940. 10.3390/diagnostics11060940 PMC8225187 34073931

[61] Joel D (2015). Sex beyond the genitalia: The human brain mosaic. Proc Natl Acad Sci U S A.

