# Supplementary material for: Differential predictability of cognitive profiles from brain structure in older males and females
Source: GeroScience. 2023 Sep 21;46(2):1713–30. doi: 10.1007/s11357-023-00934-y (PMC10828131; doi:10.1007/s11357-023-00934-y)

Supplementary Material to

Differential predictability of cognitive profiles from brain structure in older males and females

Christiane Jockwitz[1,2,*], Camilla Krämer[1,2,*], Paulo Dellani[1,2], Svenja Caspers[1,2]

[1]Institute of Neuroscience and Medicine (INM-1), Research Centre Jülich, Jülich, Germany
[2]Institute for Anatomy I, Medical Faculty & University Hospital Düsseldorf, Heinrich Heine University, Düsseldorf, Germany
*These authors contributed equally

Abbreviations

Acc.	Accuracy
DV	Demographic variables, i.e. age and education
Cond	Condition
Deconf.	Target controlled for TIV
EN	Elastic Net
F	Female subsample
folds > ref.	Percentage of folds better than reference model (dummy) in terms of R^2 (regression) or accuracy (classification)
GMV	Grey matter volume
linSVR/linSVC	Linear Support Vector Regressor/Classifier
LogReg	Logistic Regression
M	Male subsample
MAE	Mean absolute error
No-deconf.	No confound regression
r	Pearson's correlation coefficient
R^2	Coefficient of determination
RF	Random Forest
Smp	Sample
T	Target
W	Whole-sample

Supplementary Results

Supplementary Table S1. Prediction results of whole-sample cognitive profile solution based on GMV.

GMV			EN				RF				linSVR			
Smp	T	Cond	MAE	R^2	r	folds >ref.	MAE	R^2	r	folds >ref.	MAE	R^2	r	folds >ref.
W	W1	No-deconf.	0.69 (0.06)	0.12 (0.08)	0.36 (0.01)	92%	0.68 (0.06)	0.12 (0.09)	0.36 (0.01)	93%	0.69 (0.06)	0.11 (0.09)	0.36 (0.01)	94%
		Deconf.	0.78 (0.03)	0.08 (0.06)	0.28 (0.02)	86%	0.78 (0.03)	0.09 (0.07)	0.30 (0.02)	88%	0.79 (0.03)	0.07 (0.07)	0.27 (0.01)	82%
	W2	No-deconf.	0.68 (0.06)	0.05 (0.07)	0.25 (0.01)	80%	0.68 (0.06)	0.04 (0.07)	0.24 (0.01)	86%	0.68 (0.06)	0.02 (0.08)	0.22 (0.02)	73%
		Deconf.	0.79 (0.03)	0.06 (0.06)	0.25 (0.02)	88%	0.79 (0.03)	0.06 (0.05)	0.24 (0.02)	86%	0.80 (0.03)	0.03 (0.06)	0.19 (0.03)	68%
	W3	No-deconf.	0.62 (0.05)	0.03 (0.05)	0.20 (0.01)	77%	0.62 (0.05)	0.01 (0.07)	0.18 (0.02)	72%	0.61 (0.05)	0.02 (0.06)	0.19 (0.01)	69%
		Deconf.	0.80 (0.03)	0.01 (0.04)	0.13 (0.02)	68%	0.80 (0.03)	0.01 (0.05)	0.13 (0.03)	61%	0.80 (0.03)	0.00 (0.03)	0.12 (0.02)	55%
M	W1	No-deconf.	0.72 (0.08)	0.07 (0.12)	0.31 (0.02)	82%	0.72 (0.08)	0.06 (0.13)	0.30 (0.02)	80%	0.73 (0.08)	0.06 (0.14)	0.31 (0.02)	79%
		Deconf.	0.81 (0.04)	0.06 (0.09)	0.26 (0.03)	73%	0.80 (0.05)	0.07 (0.09)	0.26 (0.03)	73%	0.81 (0.05)	0.05 (0.12)	0.24 (0.03)	76%
	W2	No-deconf.	0.69 (0.09)	0.01 (0.11)	0.22 (0.01)	70%	0.68 (0.09)	0.01 (0.12)	0.22 (0.02)	66%	0.68 (0.09)	0.01 (0.13)	0.24 (0.01)	70%
		Deconf.	0.82 (0.05)	0.04 (0.07)	0.19 (0.02)	80%	0.82 (0.05)	0.03 (0.09)	0.20 (0.03)	68%	0.81 (0.05)	0.04 (0.08)	0.21 (0.01)	73%
	W3	No-deconf.	0.65 (0.08)	-0.02 (0.09)	0.14 (0.02)	62%	0.65 (0.08)	-0.04 (0.11)	0.14 (0.03)	56%	0.64 (0.08)	-0.04 (0.13)	0.16 (0.02)	55%
		Deconf.	0.81 (0.04)	0.00 (0.05)	0.09 (0.02)	62%	0.81 (0.05)	-0.01 (0.07)	0.08 (0.05)	50%	0.80 (0.05)	-0.02 (0.05)	0.12 (0.02)	57%
F	W1	No-deconf.	0.67 (0.07)	0.10 (0.11)	0.37 (0.02)	87%	0.67 (0.07)	0.11 (0.13)	0.36 (0.02)	82%	0.67 (0.07)	0.10 (0.11)	0.36 (0.02)	84%
		Deconf.	0.79 (0.05)	0.05 (0.07)	0.23 (0.02)	78%	0.79 (0.05)	0.06 (0.09)	0.25 (0.03)	76%	0.79 (0.04)	0.05 (0.06)	0.23 (0.04)	80%
	W2	No-deconf.	0.66 (0.08)	0.04 (0.12)	0.28 (0.03)	79%	0.65 (0.09)	0.05 (0.12)	0.29 (0.03)	79%	0.66 (0.08)	0.04 (0.12)	0.28 (0.02)	76%
		Deconf.	0.79 (0.04)	0.04 (0.08)	0.19 (0.05)	76%	0.79 (0.04)	0.04 (0.07)	0.21 (0.02)	74%	0.80 (0.04)	0.02 (0.05)	0.14 (0.02)	64%
	W3	No-deconf.	0.58 (0.07)	0.00 (0.10)	0.21 (0.04)	71%	0.57 (0.07)	0.02 (0.13)	0.26 (0.02)	70%	0.58 (0.08)	-0.00 (0.11)	0.20 (0.07)	67%
		Deconf.	0.79 (0.05)	0.01 (0.05)	0.12 (0.09)	70%	0.79 (0.05)	0.01 (0.09)	0.15 (0.02)	63%	0.80 (0.04)	0.00 (0.04)	0.09 (0.03)	67%

Note. Standard deviation (SD) appears in parentheses except for correlation coefficient r, for which the interquartile range (IQR) appears in parentheses.

Supplementary Table S2. Prediction results of whole-sample cognitive profile solution based on age and education (i.e. DV).

DV			EN				RF				linSVR			
Smp	T	Cond	MAE	R^2	r	folds >ref.	MAE	R^2	r	folds >ref.	MAE	R^2	r	folds >ref.
W	W1	No-deconf.	0.63 (0.05)	0.27 (0.09)	0.53 (0.00)	100%	0.64 (0.05)	0.24 (0.10)	0.51 (0.00)	99%	0.63 (0.05)	0.27 (0.10)	0.53 (0.01)	100%
		Deconf.	0.70 (0.04)	0.26 (0.08)	0.52 (0.01)	100%	0.71 (0.05)	0.24 (0.10)	0.49 (0.01)	99%	0.71 (0.05)	0.26 (0.09)	0.51 (0.01)	100%
	W2	No-deconf.	0.60 (0.05)	0.24 (0.08)	0.50 (0.00)	99%	0.60 (0.05)	0.22 (0.11)	0.49 (0.01)	98%	0.60 (0.05)	0.23 (0.10)	0.51 (0.00)	99%
		Deconf.	0.70 (0.04)	0.24 (0.08)	0.49 (0.01)	100%	0.71 (0.05)	0.22 (0.10)	0.48 (0.01)	99%	0.70 (0.05)	0.24 (0.09)	0.49 (0.01)	99%
	W3	No-deconf.	0.58 (0.05)	0.13 (0.08)	0.38 (0.00)	99%	0.58 (0.05)	0.11 (0.09)	0.36 (0.01)	94%	0.57 (0.05)	0.12 (0.08)	0.38 (0.01)	98%
		Deconf.	0.74 (0.04)	0.14 (0.07)	0.37 (0.01)	97%	0.74 (0.05)	0.11 (0.08)	0.34 (0.01)	90%	0.73 (0.04)	0.11 (0.07)	0.37 (0.01)	97%
M	W1	No-deconf.	0.63 (0.08)	0.24 (0.12)	0.52 (0.00)	98%	0.66 (0.08)	0.17 (0.15)	0.46 (0.01)	92%	0.64 (0.08)	0.23 (0.14)	0.51 (0.01)	95%
		Deconf.	0.70 (0.06)	0.25 (0.11)	0.50 (0.01)	100%	0.73 (0.06)	0.19 (0.13)	0.45 (0.02)	95%	0.70 (0.07)	0.24 (0.13)	0.50 (0.01)	93%
	W2	No-deconf.	0.62 (0.08)	0.17 (0.13)	0.46 (0.01)	95%	0.64 (0.08)	0.10 (0.17)	0.40 (0.01)	81%	0.62 (0.08)	0.16 (0.16)	0.46 (0.01)	87%
		Deconf.	0.74 (0.07)	0.20 (0.11)	0.44 (0.01)	97%	0.76 (0.07)	0.14 (0.14)	0.39 (0.02)	82%	0.74 (0.08)	0.19 (0.14)	0.44 0.01)	87%
	W3	No-deconf.	0.62 (0.07)	0.06 (0.11)	0.31 (0.01)	83%	0.63 (0.08)	0.02 (0.12)	0.26 (0.02)	69%	0.61 (0.08)	0.04 (0.14)	0.31 (0.01)	77%
		Deconf.	0.76 (0.05)	0.09 (0.09)	0.30 (0.02)	87%	0.77 (0.06)	0.05 (0.11)	0.26 (0.03)	71%	0.75 (0.06)	0.06 (0.10)	0.31 (0.02)	69%
F	W1	No-deconf.	0.62 (0.06)	0.26 (0.12)	0.54 (0.01)	97%	0.62 (0.06)	0.24 (0.15)	0.53 (0.01)	95%	0.63 (0.06)	0.24 (0.12)	0.53 (0.02)	96%
		Deconf.	0.72 (0.06)	0.25 (0.10)	0.50 (0.01)	99%	0.72 (0.07)	0.21 (0.13)	0.47 (0.02)	94%	0.72 (0.06)	0.24 (0.10)	0.49 (0.01)	99%
	W2	No-deconf.	0.57 (0.07)	0.26 (0.13)	0.55 (0.00)	95%	0.58 (0.07)	0.23 (0.15)	0.52 (0.01)	93%	0.56 (0.07)	0.26 (0.15)	0.55 (0.00)	96%
		Deconf.	0.69 (0.06)	0.26 (0.12)	0.51 (0.01)	96%	0.71 (0.07)	0.22 (0.13)	0.47 (0.01)	95%	0.69 (0.07)	0.25 (0.14)	0.51 (0.01)	94%
	W3	No-deconf.	0.54 (0.06)	0.15 (0.11)	0.44 (0.01)	90%	0.54 (0.07)	0.11 (0.16)	0.41 (0.03)	84%	0.54 (0.07)	0.15 (0.11)	0.44 (0.01)	93%
		Deconf.	0.73 (0.05)	0.16 (0.08)	0.41 (0.01)	97%	0.74 (0.07)	0.12 (0.13)	0.36 (0.03)	85%	0.73 (0.05)	0.15 (0.08)	0.40 (0.02)	97%

Note. Standard deviation (SD) appears in parentheses except for correlation coefficient r, for which the interquartile range (IQR) appears in parentheses.

Supplementary Table S3. Prediction results of whole-sample cognitive profile solution based on demographic variables and GMV (i.e. DV+ GMV).

DV+GMV			EN				RF				linSVR			
Smp	T	Cond	MAE	R^2	r	folds >ref.	MAE	R^2	r	folds >ref.	MAE	R^2	r	folds >ref.
W	W1	No-deconf.	0.62 (0.05)	0.28 (0.09)	0.54 (0.01)	100%	0.64 (0.05)	0.22 (0.09)	0.49 (0.01)	99%	0.66 (0.05)	0.18 (0.10)	0.44 (0.02)	96%
		Deconf.	0.71 (0.04)	0.25 (0.08)	0.51 (0.01)	100%	0.73 (0.04)	0.20 (0.09)	0.45 (0.01)	98%	0.75 (0.04)	0.16 (0.08)	0.40 (0.01)	96%
	W2	No-deconf.	0.60 (0.05)	0.24 (0.08)	0.51 (0.01)	99%	0.61 (0.05)	0.21 (0.09)	0.48 (0.01)	99%	0.64 (0.06)	0.13 (0.09)	0.39 (0.01)	92%
		Deconf.	0.70 (0.04)	0.24 (0.07)	0.50 (0.01)	100%	0.71 (0.04)	0.22 (0.08)	0.47 (0.01)	99%	0.75 (0.04)	0.14 (0.08)	0.37 (0.01)	92%
	W3	No-deconf.	0.58 (0.05)	0.12 (0.07)	0.36 (0.01)	95%	0.59 (0.05)	0.08 (0.07)	0.31 (0.03)	90%	0.60 (0.05)	0.04 (0.08)	0.26 (0.01)	77%
		Deconf.	0.75 (0.04)	0.12 (0.06)	0.35 (0.01)	99%	0.76 (0.04)	0.08 (0.06)	0.29 (0.02)	89%	0.78 (0.04)	0.03 (0.06)	0.22 (0.03)	76%
M	W1	No-deconf.	0.64 (0.08)	0.22 (0.12)	0.50 (0.01)	96%	0.68 (0.09)	0.13 (0.14)	0.40 (0.02)	85%	0.70 (0.09)	0.10 (0.17)	0.37 (0.03)	80%
		Deconf.	0.72 (0.05)	0.22 (0.10)	0.48 (0.02)	100%	0.75 (0.06)	0.14 (0.12)	0.37 (0.01)	86%	0.78 (0.07)	0.10 (0.14)	0.34 (0.02)	88%
	W2	No-deconf.	0.64 (0.08)	0.14 (0.11)	0.42 (0.01)	90%	0.65 (0.09)	0.12 (0.12)	0.39 (0.02)	88%	0.67 (0.09)	0.04 (0.12)	0.28 (0.01)	74%
		Deconf.	0.75 (0.06)	0.18 (0.09)	0.42 (0.01)	95%	0.77 (0.06)	0.14 (0.09)	0.37 (0.01)	94%	0.81 (0.06)	0.05 (0.10)	0.24 (0.03)	74%
	W3	No-deconf.	0.64 (0.07)	0.02 (0.10)	0.25 (0.02)	74%	0.65 (0.08)	-0.02 (0.12)	0.17 (0.02)	60%	0.64 (0.09)	-0.03 (0.14)	0.19 (0.02)	55%
		Deconf.	0.78 (0.05)	0.06 (0.07)	0.26 (0.02)	81%	0.80 (0.05)	0.01 (0.08)	0.14 (0.05)	56%	0.79 (0.05)	-0.01 (0.07)	0.14 (0.02)	50%
F	W1	No-deconf.	0.62 (0.07)	0.26 (0.12)	0.54 (0.01)	97%	0.64 (0.07)	0.20 (0.12)	0.48 (0.01)	94%	0.66 (0.07)	0.12 (0.13)	0.40 (0.03)	89%
		Deconf.	0.72 (0.06)	0.22 (0.09)	0.48 (0.01)	99%	0.75 (0.05)	0.16 (0.10)	0.39 (0.01)	95%	0.78 (0.05)	0.08 (0.08)	0.28 (0.03)	83%
	W2	No-deconf.	0.56 (0.07)	0.27 (0.12)	0.55 (0.02)	98%	0.58 (0.07)	0.23 (0.13)	0.51 (0.01)	96%	0.63 (0.08)	0.11 (0.14)	0.39 (0.02)	85%
		Deconf.	0.69 (0.05)	0.26 (0.10)	0.51 (0.02)	99%	0.72 (0.06)	0.20 (0.12)	0.45 (0.02)	94%	0.78 (0.05)	0.08 (0.09)	0.28 (0.02)	79%
	W3	No-deconf.	0.53 (0.07)	0.15 (0.10)	0.44 (0.02)	93%	0.55 (0.07)	0.11 (0.13)	0.38 (0.02)	84%	0.57 (0.08)	0.02 (0.11)	0.26 (0.02)	73%
		Deconf.	0.74 (0.05)	0.15 (0.07)	0.39 (0.02)	98%	0.75 (0.05)	0.10 (0.09)	0.32 (0.02)	86%	0.80 (0.05)	0.01 (0.07)	0.13 (0.04)	66%

Note. Standard deviation (SD) appears in parentheses except for correlation coefficient r, for which the interquartile range (IQR) appears in parentheses.

Supplementary Table S4. Prediction results of sex-specific cognitive profiles based on GMV.

GMV			EN				RF				linSVR			
Smp	T	Cond	MAE	R^2	r	folds >ref.	MAE	R^2	r	folds >ref.	MAE	R^2	r	folds >ref.
M	M1	No-deconf.	0.70 (0.09)	0.06 (0.12)	0.31 (0.01)	82%	0.70 (0.09)	0.05 (0.14)	0.30 (0.02)	75%	0.70 (0.09)	0.04 (0.13)	0.29 (0.03)	74%
		Deconf.	0.79 (0.05)	0.08 (0.08)	0.28 (0.02)	84%	0.79 (0.05)	0.07 (0.10)	0.27 (0.02)	81%	0.80 (0.04)	0.05 (0.09)	0.25 (0.04)	82%
	M2	No-deconf.	0.69 (0.08)	0.05 (0.11)	0.27 (0.03)	75%	0.69 (0.08)	0.03 (0.13)	0.26 (0.03)	75%	0.68 (0.09)	0.03 (0.14)	0.27 (0.02)	71%
		Deconf.	0.80 (0.04)	0.05 (0.07)	0.22 (0.02)	76%	0.80 (0.04)	0.04 (0.09)	0.22 (0.03)	66%	0.80 (0.05)	0.04 (0.11)	0.23 (0.03)	70%
	M3	No-deconf.	0.66 (0.08)	0.04 (0.12)	0.28 (0.02)	80%	0.66 (0.09)	0.03 (0.14)	0.27 (0.03)	72%	0.67 (0.08)	0.03 (0.12)	0.27 (0.03)	76%
		Deconf.	0.81 (0.04)	0.06 (0.07)	0.24 (0.03)	80%	0.81 (0.05)	0.06 (0.10)	0.25 (0.04)	76%	0.82 (0.04)	0.04 (0.08)	0.21 (0.04)	76%
F	F1	No-deconf.	0.68 (0.08)	0.11 (0.11)	0.38 (0.02)	89%	0.68 (0.07)	0.12 (0.13)	0.39 (0.01)	84%	0.68 (0.08)	0.10 (0.11)	0.37 (0.02)	86%
		Deconf.	0.78 (0.05)	0.06 (0.07)	0.25 (0.03)	80%	0.79 (0.05)	0.07 (0.09)	0.27 (0.02)	79%	0.78 (0.05)	0.06 (0.07)	0.24 (0.03)	80%
	F2	No-deconf.	0.67 (0.08)	0.03 (0.10)	0.25 (0.02)	75%	0.67 (0.08)	0.02 (0.12)	0.25 (0.03)	74%	0.67 (0.08)	0.02 (0.12)	0.24 (0.01)	69%
		Deconf.	0.79 (0.04)	0.03 (0.07)	0.17 (0.03)	71%	0.79 (0.04)	0.03 (0.08)	0.19 (0.05)	71%	0.80 (0.04)	0.01 (0.05)	0.12 (0.02)	58%
	F3	No-deconf.	0.64 (0.08)	0.11 (0.11)	0.38 (0.01)	91%	0.64 (0.07)	0.12 (0.13)	0.40 (0.02)	86%	0.64 (0.08)	0.11 (0.10)	0.38 (0.02)	90%
		Deconf.	0.76 (0.05)	0.07 (0.07)	0.27 (0.03)	84%	0.76 (0.06)	0.09 (0.10)	0.29 (0.02)	79%	0.76 (0.05)	0.06 (0.06)	0.26 (0.03)	85%
	F4	No-deconf.	0.61 (0.08)	0.02 (0.11)	0.25 (0.06)	72%	0.61 (0.08)	0.05 (0.14)	0.30 (0.03)	71%	0.61 (0.08)	0.04 (0.11)	0.28 (0.03)	73%
		Deconf.	0.79 (0.05)	0.02 (0.06)	0.14 (0.05)	63%	0.78 (0.05)	0.03 (0.08)	0.19 (0.03)	67%	0.80 (0.05)	0.01 (0.05)	0.12 (0.03)	63%

Note. Standard deviation (SD) appears in parentheses except for correlation coefficient r, for which the interquartile range (IQR) appears in parentheses.

Supplementary Table S5. Prediction results of sex-specific cognitive profiles based on age and education (i.e. DV).

DV			EN				RF				linSVR			
Smp	T	Cond	MAE	R^2	r	folds >ref.	MAE	R^2	r	folds >ref.	MAE	R^2	r	folds >ref.
M	M1	No-deconf.	0.60 (0.08)	0.26 (0.14)	0.55 (0.01)	98%	0.62 (0.08)	0.20 (0.17)	0.50 (0.01)	90%	0.61 (0.08)	0.25 (0.16)	0.54 (0.00)	95%
		Deconf.	0.69 (0.06)	0.28 (0.12)	0.53 (0.01)	99%	0.71 (0.07)	0.23 (0.14)	0.48 (0.02)	94%	0.69 (0.07)	0.27 (0.14)	0.53 (0.01)	98%
	M2	No-deconf.	0.62 (0.08)	0.19 (0.11)	0.47 (0.00)	96%	0.65 (0.09)	0.14 (0.15)	0.42 (0.01)	86%	0.62 (0.08)	0.19 (0.13)	0.47 (0.01)	96%
		Deconf.	0.72 (0.05)	0.21 (0.10)	0.45 (0.01)	97%	0.75 (0.06)	0.16 (0.13)	0.41 (0.01)	89%	0.73 (0.06)	0.20 (0.11)	0.45 (0.01)	96%
	M3	No-deconf.	0.58 (0.08)	0.22 (0.12)	0.50 (0.00)	98%	0.59 (0.08)	0.17 (0.15)	0.46 (0.02)	93%	0.58 (0.08)	0.21 (0.14)	0.50 (0.00)	95%
		Deconf.	0.71 (0.06)	0.24 (0.11)	0.49 (0.01)	100%	0.72 (0.06)	0.19 (0.12)	0.45 (0.03)	93%	0.71 (0.06)	0.23 (0.12)	0.49 (0.02)	98%
F	F1	No-deconf.	0.62 (0.07)	0.29 (0.13)	0.57 (0.00)	98%	0.62 (0.07)	0.26 (0.16)	0.55 (0.01)	97%	0.63 (0.07)	0.28 (0.13)	0.56 (0.01)	97%
		Deconf.	0.70 (0.07)	0.27 (0.11)	0.52 (0.01)	98%	0.71 (0.08)	0.24 (0.14)	0.49 (0.01)	93%	0.71 (0.07)	0.26 (0.11)	0.51 (0.01)	98%
	F2	No-deconf.	0.60 (0.07)	0.21 (0.13)	0.50 (0.01)	96%	0.61 (0.07)	0.18 (0.15)	0.47 (0.01)	92%	0.61 (0.07)	0.20 (0.15)	0.49 (0.01)	92%
		Deconf.	0.71 (0.05)	0.21 (0.11)	0.46 (0.01)	95%	0.73 (0.06)	0.17 (0.13)	0.42 (0.02)	92%	0.71 (0.06)	0.21 (0.13)	0.46 (0.01)	95%
	F3	No-deconf.	0.60 (0.07)	0.28 (0.12)	0.56 (0.00)	98%	0.60 (0.07)	0.22 (0.17)	0.52 (0.01)	92%	0.61 (0.07)	0.26 (0.12)	0.54 (0.01)	97%
		Deconf.	0.70 (0.06)	0.26 (0.10)	0.52 (0.01)	98%	0.71 (0.07)	0.21 (0.15)	0.50 (0.02)	92%	0.70 (0.06)	0.25 (0.11)	0.51 (0.02)	98%
	F4	No-deconf.	0.55 (0.07)	0.24 (0.12)	0.53 (0.01)	96%	0.54 (0.07)	0.22 (0.17)	0.51 (0.02)	93%	0.55 (0.07)	0.24 (0.13)	0.53 (0.01)	96%
		Deconf.	0.70 (0.06)	0.24 (0.10)	0.49 (0.01)	98%	0.71 (0.07)	0.20 (0.14)	0.46 (0.02)	94%	0.70 (0.06)	0.23 (0.10)	0.48 (0.01)	98%

Note. Standard deviation (SD) appears in parentheses except for correlation coefficient r, for which the interquartile range (IQR) appears in parentheses.

Supplementary Table S6. Prediction results of sex-specific cognitive profiles based on demographic variables and GMV (i.e. DV+ GMV).

DV+GMV			EN				RF				linSVR			
Smp	T	Cond	MAE	R^2	r	folds >ref.	MAE	R^2	r	folds >ref.	MAE	R^2	r	folds >ref.
M	M1	No-deconf.	0.63 (0.08)	0.23 (0.12)	0.52 (0.02)	99%	0.64 (0.09)	0.19 (0.14)	0.48 (0.02)	94%	0.68 (0.09)	0.10 (0.14)	0.38 (0.02)	80%
		Deconf.	0.71 (0.06)	0.26 (0.10)	0.51 (0.01)	98%	0.73 (0.07)	0.21 (0.12)	0.46 (0.01)	93%	0.77 (0.06)	0.12 (0.11)	0.36 (0.02)	83%
	M2	No-deconf.	0.63 (0.08)	0.17 (0.11)	0.45 (0.02)	94%	0.67 (0.09)	0.09 (0.13)	0.34 (0.03)	82%	0.66 (0.09)	0.08 (0.14)	0.34 (0.02)	79%
		Deconf.	0.74 (0.05)	0.18 (0.09)	0.43 (0.02)	98%	0.77 (0.06)	0.10 (0.11)	0.32 (0.03)	80%	0.77 (0.06)	0.08 (0.13)	0.30 (0.02)	79%
	M3	No-deconf.	0.60 (0.08)	0.19 (0.11)	0.46 (0.01)	94%	0.63 (0.09)	0.10 (0.14)	0.38 (0.02)	88%	0.64 (0.08)	0.09 (0.14)	0.36 (0.03)	81%
		Deconf.	0.73 (0.05)	0.20 (0.09)	0.45 (0.01)	99%	0.76 (0.06)	0.14 (0.11)	0.37 (0.02)	88%	0.79 (0.06)	0.10 (0.12)	0.33 (0.01)	83%
F	F1	No-deconf.	0.62 (0.07)	0.29 (0.12)	0.57 (0.01)	98%	0.64 (0.07)	0.22 (0.13)	0.51 (0.01)	95%	0.67 (0.07)	0.14 (0.13)	0.42 (0.01)	92%
		Deconf.	0.71 (0.06)	0.25 (0.10)	0.51 (0.01)	100%	0.74 (0.06)	0.18 (0.10)	0.42 (0.01)	95%	0.78 (0.05)	0.09 (0.09)	0.30 (0.03)	85%
	F2	No-deconf.	0.60 (0.08)	0.21 (0.12)	0.50 (0.01)	95%	0.60 (0.08)	0.19 (0.13)	0.47 (0.01)	93%	0.64 (0.08)	0.08 (0.12)	0.35 (0.02)	82%
		Deconf.	0.71 (0.05)	0.21 (0.10)	0.47 (0.02)	96%	0.72 (0.06)	0.18 (0.12)	0.42 (0.01)	94%	0.78 (0.04)	0.07 (0.09)	0.26 (0.02)	79%
	F3	No-deconf.	0.60 (0.07)	0.29 (0.11)	0.57 (0.01)	99%	0.61 (0.07)	0.21 (0.13)	0.50 (0.02)	94%	0.63 (0.08)	0.14 (0.10)	0.42 (0.00)	93%
		Deconf.	0.70 (0.06)	0.25 (0.09)	0.51 (0.01)	98%	0.73 (0.07)	0.18 (0.12)	0.42 (0.01)	89%	0.75 (0.05)	0.09 (0.07)	0.31 (0.02)	87%
	F4	No-deconf.	0.54 (0.07)	0.23 (0.11)	0.52 (0.02)	97%	0.55 (0.07)	0.21 (0.13)	0.49 (0.01)	97%	0.59 (0.09)	0.10 (0.13)	0.38 (0.04)	88%
		Deconf.	0.70 (0.06)	0.22 (0.09)	0.47 (0.02)	99%	0.71 (0.07)	0.19 (0.11)	0.43 (0.01)	96%	0.77 (0.06)	0.08 (0.08)	0.29 (0.04)	82%

Note. Standard deviation (SD) appears in parentheses except for correlation coefficient r, for which the interquartile range (IQR) appears in parentheses.

Validation analyses

Age prediction

Supplementary Table S7. Age prediction results.

	EN				RF				linSVR			
	MAE	R^2	r	folds >ref.	MAE	R^2	r	folds >ref.	MAE	R^2	r	folds >ref.
No-deconf.	4.06 (0.40)	0.39 (0.09)	0.63 (0.00)	100%	4.57 (0.38)	0.26 (0.09)	0.53 (0.00)	99%	4.00 (0.43)	0.39 (0.09)	0.63 (0.00)	100%
Deconf.	0.62 (0.05)	0.39 (0.09)	0.63 (0.01)	100%	0.70 (0.04)	0.26 (0.08)	0.51 (0.01)	100%	0.62 (0.05)	0.38 (0.10)	0.62 (0.00)	100%

Note. Standard deviation (SD) appears in parentheses except for correlation coefficient r, for which the interquartile range (IQR) appears in parentheses.

Supplementary Figure S8. Age prediction results.

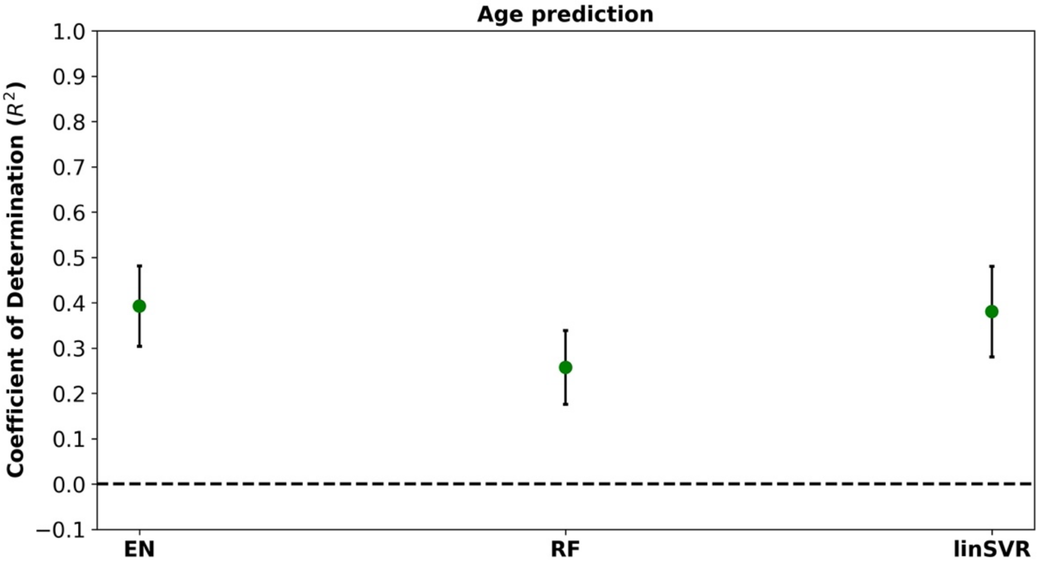

Note. Results shown for the deconf. condition.

Sex classification

Supplementary Table S9. Sex classification results.

	LogReg	folds >ref.	RF	folds > ref.	linSVC	folds > ref.
Acc. (%)	81.31 (4.45)	100%	76.19 (5.56)	100%	81.47 (4.51)	100%

Note. Standard deviation (SD) appears in parentheses.

Supplementary Figure S10. Sex classification results.

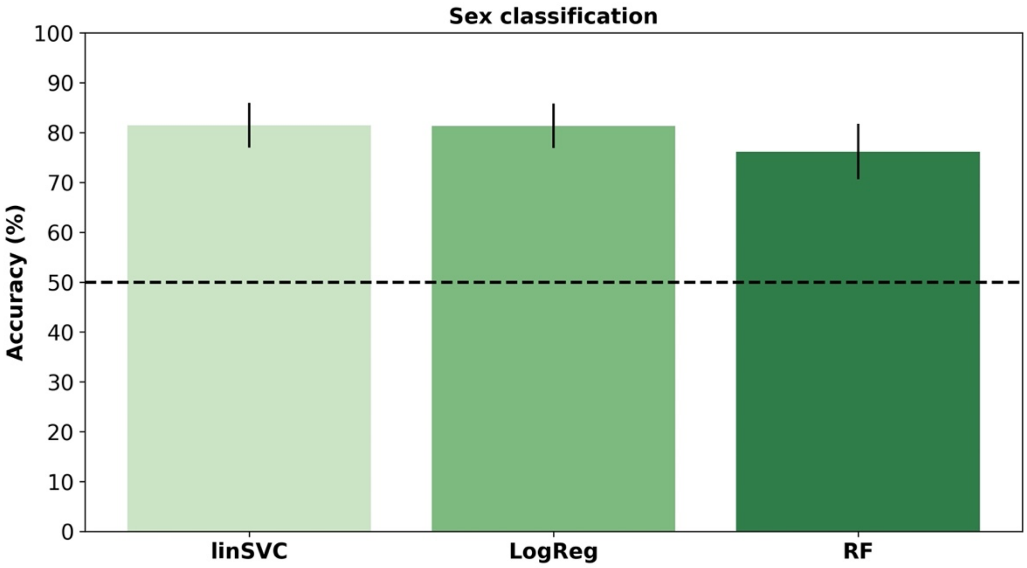

Supplement: Supplementary file 1 — Supplementary file1 (PDF 368 KB) [file 11357_2023_934_MOESM1_ESM.pdf]
